# Effect of ten-valent pneumococcal conjugate vaccine introduction on pneumococcal carriage in Fiji: results from four annual cross-sectional carriage surveys

**DOI:** 10.1016/S2214-109X(18)30383-8

**Published:** 2018-11-09

**Authors:** Eileen M Dunne, Catherine Satzke, Felisita T Ratu, Eleanor F G Neal, Laura K Boelsen, Silivia Matanitobua, Casey L Pell, Monica L Nation, Belinda D Ortika, Rita Reyburn, Kylie Jenkins, Cattram Nguyen, Katherine Gould, Jason Hinds, Lisi Tikoduadua, Joseph Kado, Eric Rafai, Mike Kama, E Kim Mulholland, Fiona M Russell

**Affiliations:** a Pneumococcal Research, Murdoch Children's Research Institute, Parkville, VIC, Australia; b Department of Paediatrics, University of Melbourne, Parkville, VIC, Australia; c Department of Microbiology and Immunology at the Peter Doherty Institute for Infection and Immunity, University of Melbourne, Parkville, VIC, Australia; d Ministry of Health and Medical Services, Suva, Fiji; e Fiji Health Sector Support Program, Suva, Fiji; f Telethon Kids Institute, Subiaco, WA, Australia; g Institute for Infection and Immunity, St George's, University of London, UK; h BUGS Bioscience, London Bioscience Innovation Centre, London, UK; i College of Medicine Nursing and Health Sciences, Fiji National University, Suva, Fiji; j London School of Hygiene & Tropical Medicine, London, UK

## Abstract

**Background:**

The indirect effects of pneumococcal conjugate vaccines (PCVs) are mediated through reductions in carriage of vaccine serotypes. Data on PCVs in Asia and the Pacific are scarce. Fiji introduced the ten-valent PCV (PCV10) in 2012, with a schedule consisting of three priming doses at 6, 10, and 14 weeks of age and no booster dose (3 + 0 schedule) without catch-up. We investigated the effects of PCV10 introduction using cross-sectional nasopharyngeal carriage surveys.

**Methods:**

We did four annual carriage surveys (one pre-PCV10 and three post-PCV10) in the greater Suva area in Fiji, during 2012–15, of 5–8-week-old infants, 12–23-month-old children, 2–6-year-old children, and their caregivers (total of 8109 participants). Eligible participants were of appropriate age, had axillary temperature lower than 37°C, and had lived in the community for at least 3 consecutive months. We used purposive quota sampling to ensure a proper representation of the Fiji population. Pneumococci were detected by real-time quantitative PCR, and molecular serotyping was done with microarray.

**Findings:**

3 years after PCV10 introduction, vaccine-serotype carriage prevalence declined, with adjusted prevalences (2015 *vs* 2012) of 0·56 (95% CI 0·34–0·93) in 5–8-week-old infants, 0·34 (0·23–0·49) in 12–23-month-olds, 0·47 (0·34–0·66) in 2–6-year-olds, and 0·43 (0·13–1·42) in caregivers. Reductions in PCV10 serotype carriage were evident in both main ethnic groups in Fiji; however, carriage of non-PCV10 serotypes increased in Indigenous Fijian infants and children. Density of PCV10 serotypes and non-PCV10 serotypes was lower in PCV10-vaccinated children aged 12–23 months than in PCV10-unvaccinated children of the same age group (PCV10 serotypes −0·56 [95% CI −0·98 to −0·15], p=0·0077; non-PCV10 serotypes −0·29 [–0·57 to −0·02], p=0·0334).

**Interpretation:**

Direct and indirect effects on pneumococcal carriage post-PCV10 are likely to result in reductions in pneumococcal disease, including in infants too young to be vaccinated. Serotype replacement in carriage in Fijian children, particularly Indigenous children, warrants further monitoring. Observed changes in pneumococcal density might be temporal rather than vaccine related.

**Funding:**

Department of Foreign Affairs and Trade of the Australian Government through the Fiji Health Sector Support Program; Victorian Government's Operational Infrastructure Support Program; Bill & Melinda Gates Foundation.

## Introduction

*Streptococcus pneumoniae* (the pneumococcus) is a common cause of community-acquired pneumonia and causes an estimated 826 000 annual paediatric deaths worldwide.^1^ Variations in the pneumococcal capsule give rise to more than 90 different serotypes, a subset of which is covered by pneumococcal conjugate vaccines (PCVs). PCVs provide direct protection against invasive pneumococcal disease, pneumonia, and the carriage of vaccine serotypes.^2^ The indirect (herd) effects of PCVs on disease are mediated by carriage, because reduced carriage of vaccine serotypes in vaccinated children limits their transmission to other age groups.^3^ Pneumococcal carriage is considered a prerequisite to pneumococcal disease.^4^ Therefore, infant immunisation extends protection to unvaccinated individuals and increases the cost-effectiveness of the vaccine.^5^, ^6^ Elimination of vaccine serotypes leads to increased colonisation and subsequent disease due to non-vaccine serotypes, known as serotype replacement.^7^ In some settings, serotype replacement in disease has partly eroded the public health benefit provided by PCVs.^8^, ^9^

Pneumococcal carriage surveys can supplement disease surveillance by assessing the effects of PCV introduction on carriage.^10^ Because the occurrence of invasive pneumococcal disease is rare, carriage surveys provide a practical alternative to disease surveillance in settings where little or no pre-PCV data on invasive pneumococcal disease are available. The effect of PCV on pneumococcal carriage varies by setting, probably because of differences in pre-PCV carriage epidemiology, vaccine schedules, and coverage.^11^ Few studies have been done in settings where PCV10 or PCV13 was introduced without previous use of PCV7. Studies assessing the effect of PCV10 in Kenya, Brazil, and Iceland (all settings without previous use of PCV7) found reductions in PCV10 serotype carriage among vaccine-eligible children and, where measured, reductions in carriage among older, unvaccinated age groups.^12^, ^13^, ^14^

**Research in context**

**Evidence before this study**

We searched PubMed for evidence of ten-valent pneumococcal conjugate vaccine (PCV10) effect on *Streptococcus pneumoniae* published before Sept 1, 2017, with the following terms in combination: “PCV10”, “*Streptococcus pneumoniae*”, “pneumococc*”, “carriage”, “colonisation”, “impact”, “effect”, “direct”, “indirect”, and “herd”. We identified three population-based studies on the effect of PCV10 on carriage in settings without previous use of PCV7; two of these studies included unvaccinated age groups. One study was done in a region of Kenya where PCV10 was introduced with a 3 + 0 schedule with a catch-up campaign for children younger than 5 years, and included participants from ten age strata. A second study was from Brazil, where PCV10 was introduced by use of a 3 + 1 schedule with catch-up for children younger than 23 months, and included only children aged 12–23 months. The third study was from Iceland, where PCV10 was introduced by use of a 2 + 1 schedule without catch up, and included children aged 1–6 years. The Kenya study reported a 64% reduction in carriage of PCV10 serotypes in children younger than 5 years and a 66% reduction in participants older than 5 years. The Brazil study found a 91% reduction in PCV10 serotype carriage in children aged 12–23 months. In Iceland, there was a 94% reduction in carriage of PCV10 serotypes in age-eligible children, and a 56% reduction in older, unvaccinated children. The Brazil and Kenya studies investigated the effect of PCV10 on *Haemophilus influenzae* carriage, with increased carriage reported in Brazil and reduced carriage reported in Kenya, although the authors of the Kenya study questioned whether the reduction they observed was attributable to the vaccine.

**Added value of this study**

We did representative population-based annual surveys investigating the effect of PCV10 introduction on pneumococcal carriage in Fiji on four age groups, providing, to our knowledge, the first published data on PCV effects in the Pacific. 3 years after PCV10 introduction, we found reductions in PCV10 serotype carriage in 12–23-month-olds, older children, and unvaccinated 5–8-week-old infants, evidence of indirect effects of PCV on carriage in young, unvaccinated infants. There was some evidence of indirect effects of PCV10 on carriage in caregivers. With sensitive and quantitative molecular methods for serotyping, we were able to investigate the effects of PCV10 introduction on circulating pneumococcal populations and on pneumococcal carriage density. We found no reduction in carriage of *H influenzae* in 12–23-month-old children after PCV10 introduction. We assessed the effects of PCV10 introduction for the two main ethnic groups in Fiji and found that, although PCV10 serotype carriage declined in both groups, serotype replacement occurred solely in the iTaukei population.

**Implications of all the available evidence**

Our study further supports the use of PCV in low-income and middle-income countries, including those in Asia and the Pacific. In addition to reducing carriage and disease caused by vaccine serotypes in vaccinated children, there is strong evidence that PCV introduction provides indirect protection. The implication of the observed effects on carriage is that PCV use could reduce pneumococcal disease in both vaccinated and unvaccinated age groups. Carriage studies are a valid and practical way to assess pneumococcal vaccine effects in settings where disease surveillance is difficult. Evidence suggests that PCV10 should not be considered a feasible strategy to reduce *H influenzae* carriage. Future research could examine how differences in PCV schedules, coverage, and the use of catch-up campaigns affect carriage in both vaccinated and unvaccinated age groups, to provide evidence to guide countries planning to introduce PCV. The speed and extent of serotype replacement in carriage, and the propensity for particular serotypes to increase after PCV introduction, varies by country and warrants ongoing surveillance. Future studies could assess the ability of models to predict serotype replacement in carriage with pre-PCV carriage data.

High pneumococcal density in the nasopharynx has been associated with respiratory infection and pneumonia in children and implicated in transmission in animal studies.^15^, ^16^ Few data on the effect of PCVs on pneumococcal carriage density exist.

Because PCV10 uses *Haemophilus influenzae* protein D as a carrier protein, there has been speculation that this vaccine could reduce *H influenzae* carriage and associated disease. The effect of PCV10 on *H influenzae* carriage has not been clearly shown in clinical trials, and results of cross-sectional studies vary.^12^, ^13^, ^17^

Support from Gavi, the Vaccine Alliance, has facilitated PCV introduction in low-income countries, although uptake has lagged in the predominantly middle-income countries in the WHO western Pacific region.^18^ Fiji has a high incidence of paediatric pneumonia (607 per 100 000 children younger than 5 years) and invasive pneumococcal disease (26·5 per 100 000 children younger than 5 years), and 44% of children aged 3–13 months carry pneumococci.^19^, ^20^, ^21^ Rates of pneumococcal carriage and disease are higher in Indigenous Fijians (iTaukei) than in Fijians of Indian descent (FID).^19^, ^20^, ^21^ In October, 2012, Fiji added PCV10 to their national immunisation programme with a schedule of three priming doses at 6, 10, and 14 weeks of age and no booster dose (3 + 0 schedule). We did annual cross-sectional nasopharyngeal carriage surveys to investigate the effect of PCV10 introduction on pneumococcal carriage by age group, year, and ethnicity. The objective of this study was to describe the prevalence of PCV10 and non-PCV10 serotypes in infants aged 5–8 weeks, children aged 12–23 months, children aged 2–6 years, and their caregivers before PCV10 introduction and during 3 years afterwards. We investigated pneumococcal carriage density by comparing PCV10-vaccinated children aged 12–23 months with unvaccinated children of the same age, hypothesising that serotype-specific immune responses in vaccinated children would restrict the ability of PCV10 serotypes to grow to high density. The use of sensitive molecular methods enabled examination of serotype distribution, pneumococcal density, and serotype-specific density. We also examined *H influenzae* carriage in the 12–23-month-old group.

## Methods

### Study design and participants

Fiji is an upper-middle-income Pacific island nation with an estimated population of 884 887, composed of 57% iTaukei and 37% FID. Approximately half of Fijians live in rural areas. This study was done in the greater Suva area. Suva is the capital and largest city in Fiji and is located in the central division of Viti Levu, the largest island in Fiji, where the majority of the population resides. We identified communities and health centres on the basis of proximity to ultra-low temperature freezers, because carriage samples needed to be stored within 8 h of collection. Recruitment occurred at the two largest health centres in the greater Suva area, Nausori Health Centre and Valelevu Health Centre, and surrounding communities. Recruitment and carriage surveys were done in 2012–15 during July–December, in the same villages and health centres each year (appendix). Approximately 500 participants were enrolled each year from each of the following age groups: 5–8-week-old infants, 12–23-month-old children, 2–6-year-old children, and their caregivers. We calculated sample size on the basis of an anticipated 50% decrease in PCV10 serotype carriage prevalence from an estimated baseline prevalence of 16%, and increased size to 500 per group because the true baseline and effect sizes were unknown. Purposive quota sampling was undertaken, so that a representative sample of the Fiji population was included, with enrolment targets of 60% iTaukei and 40% FID and approximately 50% of participants from rural areas. Inclusion criteria were appropriate age, axillary temperature lower than 37°C, and having lived in the community for at least 3 consecutive months.

Written informed consent was obtained (from parent or guardian for paediatric participants). This study was done according to the protocol approved by the Fiji National Health Research and Ethics Review Committee (201228) and the University of Melbourne Health Sciences Human Ethics SubCommittee (1238212).

### Study procedures and laboratory analyses

Study staff recorded information on demographics and potential risk factors for pneumococcal carriage on a data collection form. PCV10 status was obtained from maternal and child health cards or the child's health centre record, if the card was not available. Nasopharyngeal swabs were collected and stored according to WHO guidelines.^22^ Detection of pneumococcus and *H influenzae* was done by real-time quantitative PCR (qPCR) targeting the *lytA* gene for pneumococcus and *hpd* gene for *H influenzae*.^23^, ^24^ Molecular serotyping of pneumococci by microarray was done as previously described in the literature (more details in the appendix).^25^

### Statistical analysis

We did statistical analyses with GraphPad Prism (version 7.03) and Stata (versions 14.2 and 15.1). The χ^2^ test was used to compare categorical data, and the Kruskal–Wallis test to compare continuous data, unless otherwise noted. Bacterial density data were log_10_ transformed and reported as log_10_ genome equivalents per mL (log_10_ GE per mL). Because not all density data were normally distributed, we used non-parametric analysis methods.

We assessed carriage prevalence of overall pneumococci, PCV10 serotypes, and non-PCV10 serotypes for each study year. We calculated prevalence ratios by comparing each post-introduction year with 2012, the pre-PCV10 introduction year. For each participant and age group, univariable log-binomial regression models were fitted to estimate unadjusted prevalence rate ratios of overall pneumococcal carriage in association with participant characteristics. With use of variables considered associated with overall carriage (p<0·1), multivariable log-binomial regression models were fitted to estimate the adjusted prevalence ratios of overall pneumococcal, PCV10 serotype, and non-PCV10 serotype carriage. The month of swab collection was also included in the adjusted models. Separate models were fitted for each age group. When log-binomial models did not converge, we used Poisson models with robust 95% CIs, with incidence ratios as a proxy for prevalence ratios.^12^ The comparison of log-binomial results with Poisson results where possible confirmed the comparability. Percent reductions in PCV10 carriage were quantified as (1–adjusted prevalence ratio) × 100.

To investigate the potential effects of PCV10 introduction on pneumococcal density, we focused on the 12–23-month-old age group, because we anticipated that any vaccine-related effects primarily would be in the PCV10 age-eligible group. We used quantile (median) regression to analyse pneumococcal density by PCV10 vaccination status for 12–23-month-old children, combining data from all 4 years. To adjust for potential confounders, a multivariable quantile regression model included variables found to be associated with density by univariable analysis (p<0·1).

### Role of the funding source

Fiji Health Sector Support Programme contributed to study conception and design. The funders had no role in data collection, analysis, or interpretation, or decision to publish. EMD had full access to study data and FMR had final responsibility for the decision to submit for publication.

## Results

There were 8109 participants in the surveys, with characteristics shown in table 1. The ethnic distribution of participants was similar across surveys, with minor variations in residential location. A total of 43 participant samples were excluded from analysis because of insufficient volume, sample loss, labelling errors, or other technical issues. In the 12–23 months age group, the percentage of participants who were PCV10 vaccinated increased from zero in 2012 and 11 (2%) in 2013, to 474 (95%) in 2014 and 498 (100%) in 2015. By contrast, for 2–6-year-olds, 116 (23%) participants were vaccinated in 2015 (table 1).

**Table 1:** Characteristics of study participants in annual cross-sectional pneumococcal carriage surveys in Fiji, by age group and year

		**2012**	**2013**	**2014**	**2015**	p value*
**5–8-week-old infants**						
Number of participants		499	510	500	497	..
Median age in weeks (IQR)		6·3 (5·6–7·1)	5·7 (5·3–5·9)	6·3 (6·1–6·6)	6·1 (5·6–6·6)	0·450
Sex						
	Girls	258 (52%)	251 (49%)	219 (44%)	248 (50%)	0·074
	Boys	241 (48%)	259 (51%)	281 (56%)	249 (50%)	..
Ethnicity						
	iTaukei	298 (60%)	310 (61%)	291 (58%)	303 (61%)	0·923
	FID	199 (40%)	197 (39%)	207 (41%)	193 (39%)	..
	Other	2 (<1%)	3 (1%)	2 (<1%)	1 (<1%)	..
Residential location						
	Rural	244 (49%)	259 (51%)	251 (50%)	281 (57%)	0·052
	Urban or peri-urban	255 (51%)	251 (49%)	249 (50%)	216 (43%)	..
Median number of children younger than 5 years in household (IQR)		2 (1–2)	1 (1–2)	1 (1–2)	2 (1–2)	0·882
Exposure to household cigarette smoke		203 (41%)	267 (52%)	265 (53%)	276 (56%)	<0·0001
Symptoms of URTI		55 (11%)	37 (7%)	108 (22%)	111 (22%)	<0·0001
Antibiotic use in previous fortnight†		12 (2%)	9 (2%) (n=509)	15 (3%)	6 (1%)	0·221
Poverty‡		308 (69%) (n = 447)	222 (44%)	186 (37%)	278 (56%)	<0·0001
Born by vaginal delivery		456 (91%)	457 (90%)	405 (81%)	424 (85%)	<0·0001
Breastfeeding at time of survey		468 (94%) (n=498)	448 (88%)	472 (94%)	469 (94%)	<0·0001
**12–23-month-old children**						
Number of participants		500	505	501	498	..
Median age in months (IQR)		16·6 (14·3–19·7)	18·3 (14·9–21·0)	17·2 (14·5–20·0)	18·2 (15·1–21·5)	0·0001
Sex						
	Girls	239 (48%)	231 (46%)	201 (40%)	230 (46%)	0·079
	Boys	261 (52%)	274 (54%)	300 (60%)	268 (54%)	..
Ethnicity						
	iTaukei	300 (60%)	302 (60%)	291 (58%)	301 (60%)	0·134
	FID	197 (39%)	203 (40%)	210 (42%)	197 (40%)	..
	Other	3 (1%)	0 (0%)	0 (0%)	0 (0%)	..
Residential location						
	Rural	243 (49%)	255 (50%)	251 (50%)	201 (40%)	0·0035
	Urban or peri-urban	257 (51%)	250 (50%)	250 (50%)	297 (60%)	..
Median number of children younger than 5 years in household (IQR)		2 (1–2)	2 (1–2)	1 (1–2)	2 (1–3)	0·0001
Exposure to household cigarette smoke		262 (52%)	284 (56%)	303 (60%)	247 (50%)	0·0036
Symptoms of URTI		165 (33%)	175 (35%)	224 (45%)	183 (37%)	0·0006
Previously vaccinated with PCV10		0 (0%)	11 (2%) (n=504)	474 (95%)	498 (100%)	<0·0001
Antibiotic use in previous fortnight†		53 (11%)	30 (6%) (n=503)	12 (2%)	28 (6%)	<0·0001
Poverty‡		341 (77%) (n=441)	307 (61%)	253 (51%) (n=500)	258 (52%)	<0·0001
Breastfeeding at time of survey		187 (37%)	86 (17%)	237 (47%)	141 (28%)	<0·0001
**2–6-year-old children**						
Number of participants		518	516	505	513	..
Median age in years (IQR)		3·8 (2·8–5·2)	3·8 (3·0–4·7)	4·1 (3·1–5·0)	4·1 (3·1–5·2)	0·028
Sex						
	Girls	250 (48%)	234 (45%)	253 (50%)	266 (52%)	0·190
	Boys	268 (52%)	282 (55%)	252 (50%)	247 (48%)	..
Ethnicity						
	iTaukei	313 (60%)	305 (59%)	298 (59%)	303 (59%)	0·636
	FID	197 (38%)	208 (40%)	203 (40%)	207 (40%)	..
	Other	8 (2%)	3 (1%)	4 (1%)	3 (1%)	..
Residential location						
	Rural	260 (50%)	260 (50%)	249 (49%)	243 (47%)	0·759
	Urban or peri-urban	258 (50%)	256 (50%)	256 (51%)	270 (53%)	..
Median number of children younger than 5 years in household (IQR)		2 (1–2)	2 (1–2)	2 (1–2)	2 (1–2)	0·261
Exposure to household cigarette smoke		308 (59%)	296 (57%)	251 (50%)	274 (53%)	0·0090
Symptoms of URTI		203 (39%)	155 (30%)	141 (28%)	157 (31%)	0·0005
Previously vaccinated with PCV10		0 (0%)	0 (0%)	1 (<1%)	116 (23%)	<0·0001
Antibiotic use in previous fortnight†		61 (12%)	27 (5%) (n=515)	9 (2%)	11 (2%)	<0·0001
Poverty‡		342 (78%) (n=437)	354 (69%)	295 (58%)	266 (52%)	<0·0001
**Caregivers**						
Number of participants		508	511	516	512	..
Median age in years (IQR)		30·5 (25·2–38·6)	32·0 (25·5–40·5)	32·0 (25·6–39·3)	32·1 (25·9–44·7)	0·107
Sex						
	Women	464 (91%)	449 (88%)	447 (87%)	443 (87%)	0·061
	Men	44 (9%)	62 (12%)	69 (13%)	69 (13%)	
Ethnicity						
	iTaukei	310 (61%)	309 (60%)	296 (57%)	305 (60%)	0·330
	FID	196 (39%)	202 (40%)	215 (42%)	205 (40%)	..
	Other	2 (<1%)	0 (0%)	5 (1%)	2 (<1%)	..
Residential location						
	Rural	239 (47%)	257 (50%)	216 (42%)	243 (47%)	0·054
	Urban or peri-urban	269 (53%)	254 (50%)	300 (58%)	269 (53%)	..
Median number of children younger than 5 years in household (IQR)		1 (1–2)	1 (1–2)	1 (1–2) (n=513)	1 (1–2)	0·545
Exposure to household cigarette smoke		276 (54%)	276 (54%)	269 (52%)	296 (58%)	0·323
Symptoms of URTI		106 (21%)	82 (16%)	84 (16%)	106 (21%)	0·064
Antibiotic use in previous fortnight†		38 (7%)	19 (4%)	11 (2%)	16 (3%)	0·0001
Poverty‡		306 (71%) (n=433)	345 (68%)	280 (55%) (n=510)	258 (50%)	<0·0001

Data are n (%), unless otherwise specified. In 2012, there was some missing data for poverty, likely due to perceived sensitivity when asking about income; this was rectified by additional field training in subsequent surveys. FID=Fijians of Indian descent. URTI=upper respiratory tract infection (includes any of the following: ear discharge, runny nose, and cough at time of survey). PCV10=received two or three doses of ten-valent pneumococcal conjugate vaccine before the survey; no 5–8-week-old infants or caregivers received this vaccine.

* p values compare across years using chi-squared test for categorical data and Kruskal-Wallis test for continuous data.

† As reported by parent or guardian.

‡ Poverty defined as weekly family income below the basic needs poverty line (<FJ$175 per week).^26^

**Figure 1:**
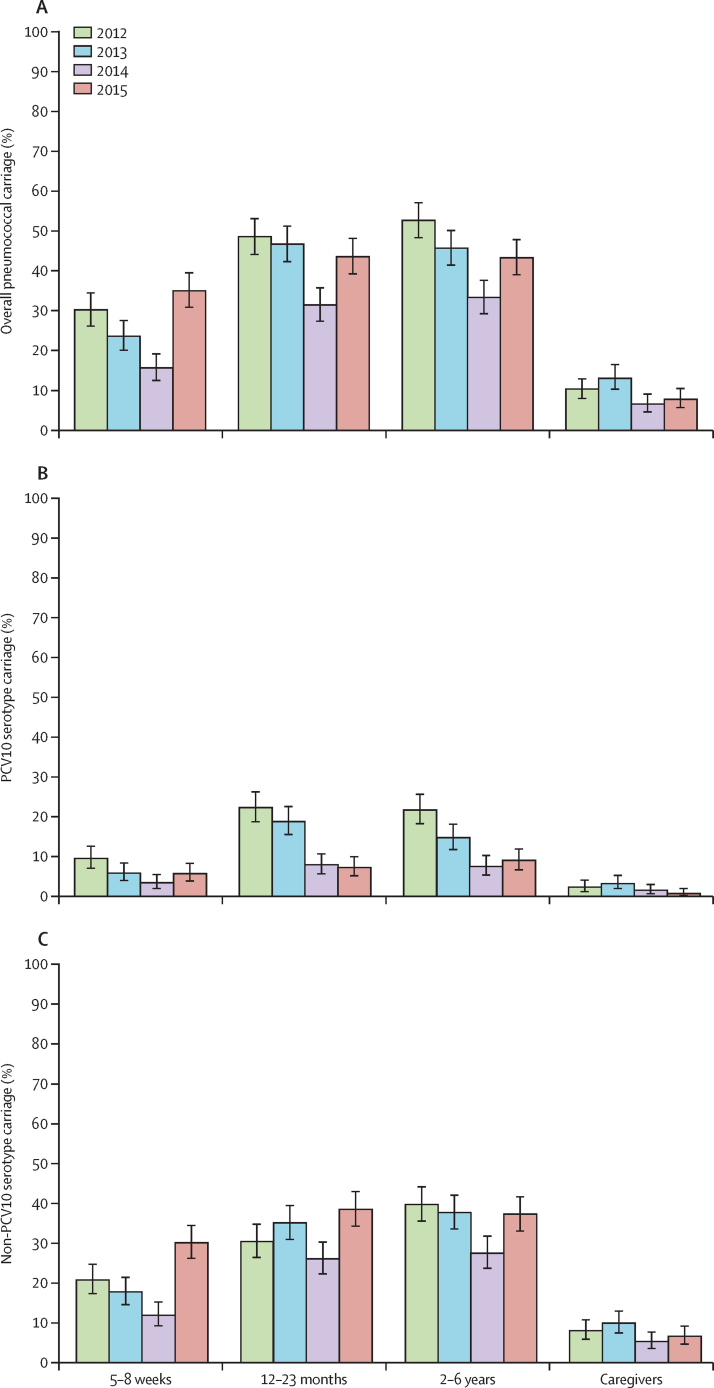
Nasopharyngeal carriage prevalence per age group and year Carriage prevalence of overall pneumococci (A), ten-valent pneumococcal conjugate vaccine (PCV10) serotype pneumococci (B), and non-PCV10 serotype pneumococci (C) in four different age groups in Fiji, in 2012–15. PCV10 was introduced in 2012 after our first carriage survey was done. Error bars depict 95% CI.

Figure 1 and appendix (p 3) show the carriage prevalence of overall pneumococci, PCV10 serotypes, and non-PCV10 serotypes, by age group and year. Table 2 shows the unadjusted and adjusted prevalence ratios for each post-PCV10 year compared with 2012. 3 years after PCV10 introduction, reductions in PCV10 serotype carriage were observed in the three paediatric groups (table 2). Because 116 (23%) of children aged 2–6 years were PCV10 vaccinated in 2015, we did a post-hoc subanalysis including only the unvaccinated children to investigate indirect effects specifically. The adjusted prevalence ratio of PCV10 serotype carriage for these unvaccinated children was 0·52 (95% CI 0·37–0·75, p=0·0004). In caregivers, there were no significant changes in carriage of PCV10 serotypes. Carriage of non-PCV10 serotypes increased significantly in 5–8-week-olds and 12–23-month-olds in 2015.

**Table 2:** Unadjusted (PR) and adjusted (aPR) carriage prevalence ratios for all pneumococci, ten-valent pneumococcal conjugate vaccine (PCV10) serotypes, and non-PCV10 serotypes compared with 2012

	**2013**		**2014**		**2015**	
	PR (95% CI)	aPR* (95% CI)	PR (95% CI)	aPR* (95% CI)	PR (95% CI)	aPR* (95% CI)
**All pneumococci**						
5–8 weeks†	0·78 (0·64–0·96)	0·89 (0·68–1·15)	0·52 (0·40–0·66)	0·69 (0·51–0·95)	1·17 (0·98–1·40)	1·29 (1·05–1·58)
12–23 months†	0·96 (0·84–1·09)	0·78 (0·68–0·90)	0·65 (0·55–0·76)	0·64 (0·54–0·76)	0·90 (0·79–1·03)	0·91 (0·79–1·05)
2–6 years†	0·87 (0·77–0·98)	0·85 (0·73–0·99)	0·63 (0·54–0·73)	0·69 (0·59–0·81)	0·82 (0·72–0·93)	0·90 (0·79–1·03)
Caregivers	1·26 (0·90–1·76)	1·23 (0·81–1·86)	0·63 (0·42–0·95)	0·68 (0·43–1·07)	0·75 (0·51–1·11)	0·82 (0·54–1·23)
**PCV10 serotypes**						
5–8 weeks	0·62 (0·40–0·96)	0·44 (0·26–0·77)	0·36 (0·21–0·62)	0·34 (0·18–0·66)	0·61 (0·39–0·96)	0·56 (0·34–0·93)
12–23 months	0·84 (0·66–1·08)	0·63 (0·48–0·83)	0·36 (0·25–0·50)	0·37 (0·26–0·52)	0·33 (0·23–0·47)	0·34 (0·23–0·49)
2–6 years	0·68 (0·52–0·88)	0·56 (0·40–0·79)	0·35 (0·25–0·49)	0·35 (0·24–0·52)	0·42 (0·30–0·58)	0·47 (0·34–0·66)
Caregivers	1·41 (0·68–2·92)	1·24 (0·47–3·30)	0·65 (0·27–1·59)	0·76 (0·26–2·17)	0·33 (0·11–1·02)	0·43 (0·13–1·42)
**Non-PCV10 serotypes**						
5–8 weeks†	0·85 (0·65–1·09)	1·16 (0·85–1·60)	0·58 (0·43–0·78)	0·92 (0·63–1·34)	1·46 (1·17–1·82)	1·72 (1·33–2·22)
12–23 months	1·15 (0·96–1·38)	1·09 (0·90–1·32)	0·86 (0·70–1·05)	0·91 (0·74–1·12)	1·27 (1·06–1·51)	1·28 (1·07–1·54)
2–6 years†	0·95 (0·81–1·11)	0·99 (0·82–1·20)	0·69 (0·58–0·83)	0·78 (0·65–0·95)	0·94 (0·80–1·10)	1·05 (0·89–1·23)
Caregivers	1·24 (0·83–1·83)	1·28 (0·80–2·06)	0·67 (0·42–1·07)	0·74 (0·45–1·23)	0·83 (0·53–1·28)	0·90 (0·57–1·43)

* Variables were adjusted for each age group. 5–8 weeks: ethnicity, two or more children younger than 5 years in the household (multiple under-5s), symptoms of upper respiratory tract infection (URTI), poverty, method of delivery, breastfeeding status, and month of swab collection. 12–23 months: ethnicity, residential location, multiple under-5s, URTI, poverty, and month of swab collection. 2–6 years: ethnicity, residential location, multiple under-5s, exposure to household cigarette smoke, URTI, poverty, and month of swab collection. Caregivers: ethnicity, sex, multiple under-5s, URTI, poverty, and month of swab collection.

† Log-binomial regression model data did not converge, so a Poisson regression model was used to estimate adjusted prevalence ratios.

**Figure 2:**
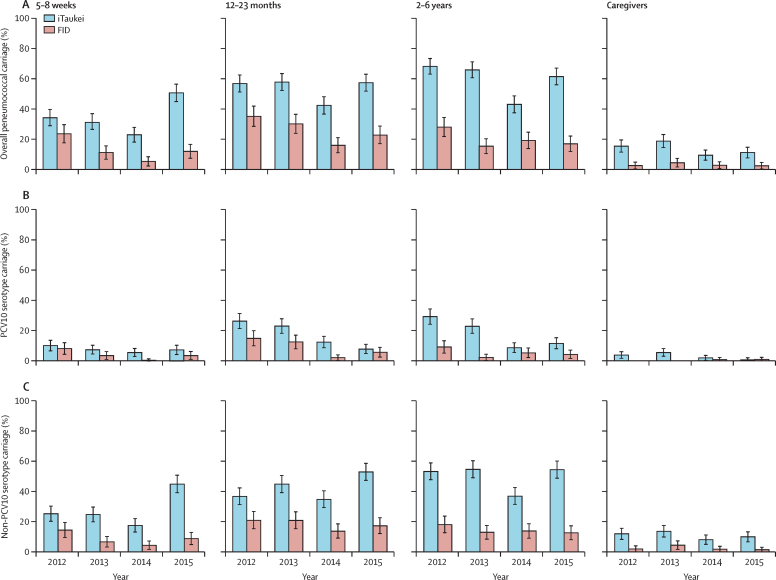
Nasopharyngeal carriage prevalence in the main Fiji ethnic groups Nasopharyngeal carriage prevalence of overall pneumococci (A), ten-valent pneumococcal conjugate vaccine (PCV10) serotype pneumococci (B), and non-PCV10 serotype pneumococci (C) in Indigenous Fijians (iTaukei) and Fijians of Indian descent (FID) by year in four different age groups. Error bars depict 95% CI.

Pneumococcal carriage by ethnicity is shown in figure 2 (see appendix, pp 4–6, for prevalence ratios). Pneumococcal carriage was higher in iTaukei participants than in FID participants. For paediatric groups, reductions in PCV10 serotype carriage in both ethnicities were consistent with overall results. In iTaukei caregivers, PCV10 serotype carriage was lower in 2015 than in 2012 (appendix, p 4). No FID caregivers carried a PCV10 serotype in 2012, therefore prevalence ratios were not calculated. An increase in carriage prevalence of non-PCV10 serotypes in paediatric groups was observed only in iTaukei participants (figure 2).

**Figure 3:**
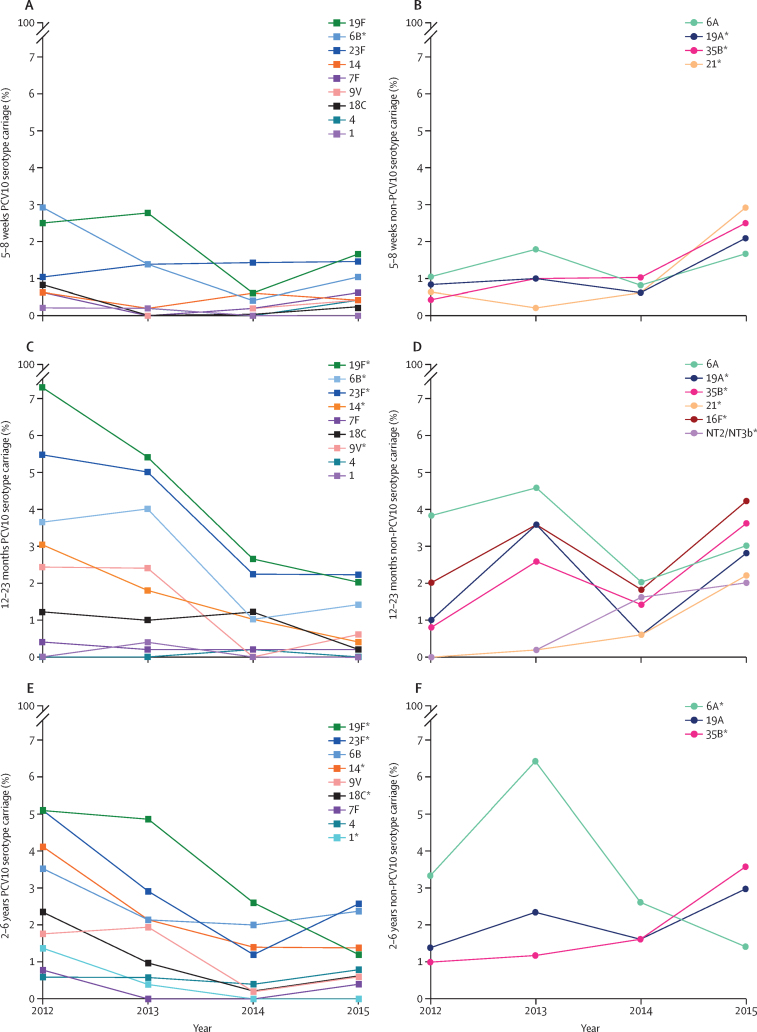
Nasopharyngeal carriage prevalence of pneumococcal serotypes Carriage prevalence of pneumococcal serotypes in Fijian children aged 5–8 weeks (A and B), 12–23 months (C and D), and 2–6 years (E and F). (A), (C), and (E) shown ten-valent pneumococcal conjugate vaccine (PCV10) serotypes. (B), (D), and (F) show vaccine-related serotypes 6A and 19A and any non-PCV10 serotypes that significantly increased in 2015, compared with 2012 (appendix p 10). *p<0·05 (exact p values shown in appendix, p 10).

We identified 3124 pneumococci, belonging to 54 capsular serotypes plus seven genetic variants of non-encapsulated pneumococci. 19·8% (95% CI 18·2–21·4) of pneumococcal-positive samples contained more than one serotype. The proportion of pneumococci that belonged to PCV10 serotypes declined significantly for all age groups in 2015, and for the 12–23 months and 2–6 years groups in 2013, and 2014, compared with proportions in 2012 (appendix p 7). Serotype-specific carriage prevalences for paediatric groups are shown in figure 3 and the appendix (p 10). Carriage of several PCV10 serotypes was lower in 2015 than in 2012. Serotype 19A increased in 5–8-week-olds and 12–23-month-olds, and serotype 35B increased in all three paediatric groups. We did not examine serotype-specific prevalences in caregivers because of the small values in this category. Changes in the serotype distribution of the overall pneumococcal population are shown in the appendix (p 9).

**Figure 4:**
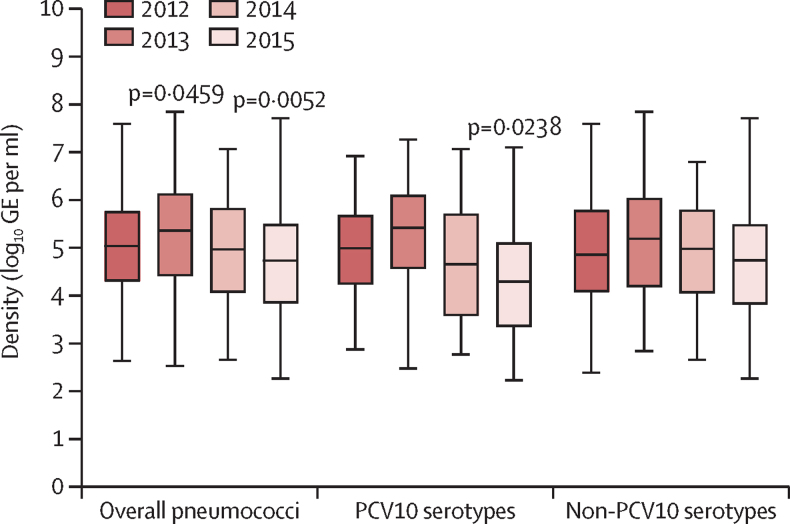
Nasopharyngeal carriage density in children aged 12–23 months Carriage density (log_10_ genome equivalents [GE] per mL) of overall pneumococci, ten-valent pneumococcal conjugate vaccine (PCV10) serotypes, and non-PCV10 serotypes in children aged 12–23 months. Boxes show IQR with a central line denoting the median, and lines extend 1·5 times IQR past the quartiles. With Kruskal–Wallis test, we found that median density varied among years for overall pneumococci (p<0·0001), PCV10 serotypes (p=0·0002), and non-PCV10 serotypes (p=0·0040). Dunn's post-test was used for p values (compared with 2012).

Pneumococcal carriage density varied over time (figure 4). Density of PCV10 serotypes was lower in 2015 (median 4·29 log_10_ GE per mL, IQR 3·35–5·10), than in 2012 (4·98 log_10_ GE per mL, 4·23–5·67; p=0·0238 [Dunn's post-test]). There were no differences in pneumococcal carriage density between iTaukei children (median 5·05 log_10_ GE per mL, IQR 4·18–5·82) and FID children (4·97 log_10_ GE per mL, 4·11–5·72; p=0·531). The densities of overall pneumococci, PCV10 serotypes, and non-PCV10 serotypes were lower in PCV10-vaccinated 12–23-month-old children than in unvaccinated children of the same age group (table 3). We found no significant differences in the density of individual serotypes.

**Table 3:** Quantile (median) regression analysis of pneumococcal density in ten-valent pneumococcal conjugate vaccine (PCV10) vaccinated and unvaccinated children aged 12–23 months who were pneumococcal carriers (n=848)

	**Number of carriers**	Median density*	**Adjusted coefficient**†**(95% CI)**	**p value**	**Pseudo-R^2^ value**
**Overall pneumococci**					
Unvaccinated	485	5·16	Reference	0·0004	0·031
PCV10-vaccinated	363	4·85	−0·39 (−0·61 to −0·18)	..	..
**PCV10 serotypes**					
Unvaccinated	207	5·16	Reference	0·0077	0·064
PCV10-vaccinated	72	4·45	−0·56 (−0·98 to −0·15)	..	..
**Non-PCV10 serotypes**					
Unvaccinated	331	5·03	Reference	0·0334	0·018
PCV10-vaccinated	312	4·86	−0·29 (−0·57 to −0·02)	..	..

Data are from all four years combined. PCV10-vaccinated=received one or more doses of PCV10 vaccine.

* Density reported in log_10_ genome equivalents per mL.

† Coefficient is the difference in medians as determined by quantile regression, adjusted for upper respiratory tract infection symptoms, poverty, and two or more children younger than 5 years in the household.

We did not observe a significant difference in carriage prevalence of *H influenzae* in the 12–23 months age group between 2012 (39·6%, 95% CI 35·3–44·4) and 2015 (40·0%, 35·6–44·4; p=0·84).

## Discussion

To our knowledge, this study provides the first evidence of a population effect of PCV introduction in a low-income or middle-income country in the Asia-Pacific region. We observed direct and indirect effects on pneumococcal carriage, including reduced PCV10 serotype carriage in infants too young to be vaccinated. We expect that this reduction in vaccine-type carriage will translate to reductions in pneumococcal disease, as seen in settings with robust disease surveillance. Findings from a prospective population-based study^27^ in the USA showed a 39% decline in neonatal invasive pneumococcal disease and a 45% decline in invasive pneumococcal disease in the 31–60 days age group after PCV7 introduction. Surveillance data^28^ from South Africa, covering pre-PCV, post-PCV7, and post-PCV13 periods showed that the incidence of invasive pneumococcal disease declined by 36% in infants younger than 10 weeks. In Israel,^29^ reductions in pneumococcal otitis media were observed in infants younger than 4 months after PCV13 introduction; because this age group received one vaccine dose or none, protection would largely be indirect.

2 years after PCV10 introduction, we observed a 63% reduction in PCV10 serotype carriage in children aged 12–23 months similar to the 64% reduction in vaccine-eligible age groups observed in Kenya.^12^ Reductions in carriage of PCV10 serotypes in children aged 12–23 months would be due to a combination of both direct and indirect effects.

We found no difference in carriage of *H influenzae* in vaccinated age groups pre-introduction and post-introduction of PCV10, consistent with the results from a clinical trial in Finland.^30^ In 12–23-month-old children in Brazil,^13^ carriage of non-typeable *H influenzae* strains (NTHi) increased after PCV10 introduction. In Kenya,^12^ NTHi carriage in children younger than 5 years declined in the post-PCV10 period, but the authors did not see evidence of an effect when comparing vaccinated with unvaccinated children. Together, these results suggest that PCV10 does not reduce carriage of *H influenzae*.

We observed potential indirect effects in Fiji, 3 years after PCV10 introduction in young, unvaccinated infants and older children, even in the absence of a catch-up campaign or a booster dose. Most Gavi-supported countries use a 3 + 0 schedule for PCV, therefore our results are relevant for these settings. The degree of indirect effects is likely to be associated with the coverage achieved.^31^ The results of this study are consistent with a systematic review that found that carriage of vaccine serotypes declined in non-target age groups after PCV introduction.^11^ Reductions in vaccine-type carriage occurred contemporaneously with reductions in vaccine-type invasive pneumococcal disease, supporting the usefulness of carriage as an alternative for disease surveillance to show the population effects of PCV.^11^

In this study, we found evidence of indirect effects in caregivers, but only in iTaukei individuals, probably due to the low baseline carriage of PCV10 serotypes in FID individuals. Similarly, a non-significant decline in PCV13 serotype carriage was reported in adult Native Americans,^32^ which was attributed to a low baseline carriage (2%).

Pneumococcal carriage rates are consistently higher in iTaukei than in FID individuals, as observed in this study and in previous studies.^21^, ^33^ After PCV10 introduction, carriage of PCV10 serotypes declined in both ethnic groups. However, serotype replacement in carriage occurred in iTaukei children and infants only, possibly because of higher baseline carriage of non-PCV10 serotypes. Invasive pneumococcal disease surveillance is ongoing, because serotype replacement in carriage might become more prominent and lead to serotype replacement in disease. Generally, PCV use decreases—but does not eliminate—disparity in invasive pneumococcal disease rates among different ethnic populations living in the same area.^34^

In many settings, serotype 19A was the primary replacement serotype after PCV7 introduction and was associated with increases in invasive pneumococcal disease.^35^ In Fiji, carriage of 19A increased in young children. However, this increase was small and similar in magnitude to several other non-PCV10 serotypes. In the PCV10 studies in Brazil^13^ and Iceland,^14^ carriage of 19A did not change after PCV10 introduction. In Kenya, 5 years after PCV10 introduction, 19A carriage prevalence had increased among children younger than 5 years.^36^ Consistent with other studies,^12^, ^13^, ^14^, ^30^ we saw no difference in carriage of 6A indicative of a cross-protective effect of PCV10.

The laboratory methods used in this study enabled quantitative detection of multiple serotypes within the same sample, a common occurrence in Fiji. Future studies might consider if and how multiple serotype carriage influences vaccine effect, and how results compare with those obtained with traditional methods.

The density of both PCV10 and non-PCV10 serotypes was lower in vaccinated children than in unvaccinated children, although we had hypothesised that vaccination would primarily affect PCV10 serotypes. Because most samples from unvaccinated children were from the early PCV10 introduction period (2012–13), and samples from vaccinated children from the later PCV10 period (2014–15), differences in density might be due to fluctuations in other unmeasured confounders rather than related to PCV10. In a study^37^ done with semi-quantitative methods, Dagan and colleagues found no difference in the density of the six additional serotypes included in PCV13 when comparing densities in children who received PCV7 with those in children vaccinated with PCV13.

In settings such as Fiji, carriage surveys are a practical way of assessing the effect of PCV introduction. A reduction in vaccine serotype carriage in the community is likely to translate to a reduction in vaccine-type pneumococcal disease. A limitation of any observational study on this topic is that it is difficult to distinguish vaccine effects from secular trends. Pneumococcal carriage prevalence in 2014 was low across all age groups, despite each annual survey being done with the same study methods and procedures. The decline in PCV10 serotypes might be associated with high vaccine coverage. However, this does not explain the decline in non-PCV10 serotypes, which might be related to unknown, and unmeasured, confounders. In 2015, a local influenza outbreak^38^ coincided with the carriage survey, which might have affected pneumococcal carriage that year. Such examples highlight the complexity of trend analyses. We did one carriage survey before PCV10 introduction, whereas multiple years of pre-PCV data would provide a more robust baseline.

PCV10 coverage in study participants was very high and, although consistent with national coverage rates (89% for the third dose of PCV10 in 2015), it might not be representative of remote areas of Fiji.^39^ Study participants could belong to the same household, but our analyses did not account for this lack of independence. Older children and adolescents were not surveyed and might represent a reservoir for vaccine-serotype carriage after PCV introduction.^40^

In conclusion, we have shown that the carriage of PCV10 serotypes in both vaccinated and unvaccinated individuals declined after PCV10 introduction in Fiji. Importantly, indirect effects were observed in infants too young to be vaccinated as well as older age groups who did not receive the vaccine. Serotype replacement in carriage was beginning to emerge 3 years after PCV10 introduction and occurred solely in iTaukei children. We found no effect of PCV10 introduction on carriage of *H influenzae*. Monitoring of changes in pneumococcal carriage continues to be a valuable way to identify population effects after PCV10 introduction.
