# Supplementary material for: Effect of ten-valent pneumococcal conjugate vaccine introduction on pneumococcal carriage in Fiji: results from four annual cross-sectional carriage surveys
Source: Lancet Glob Health. 2018 Nov 9;6(12):e1375–85. doi: 10.1016/S2214-109X(18)30383-8 (PMC6231327; doi:10.1016/S2214-109X(18)30383-8)
Supplement: Supplementary appendix [file mmc1.pdf]

THE LANCET
Global Health

Supplementary appendix

This appendix formed part of the original submission and has been peer reviewed. We post it as supplied by the authors.

Supplement to: Dunne EM, Satzke C, Ratu FT. Effect of ten-valent pneumococcal conjugate vaccine introduction on pneumococcal carriage in Fiji: results from four annual cross-sectional carriage surveys. *Lancet Glob Health* 2018; **6:** e1375–85.

Supplementary Information

Detailed methods

Study design and enrolment

The Republic of Fiji is comprised of >300 islands in the South Pacific Ocean. The main island of Viti Levu has 81% of the population. Fiji is divided into four Divisions. Suva, the capital of Fiji, lies within the Central Division. The Greater Suva area, including the surrounding towns of Lami, Nasinu, and Nausori comprises over one-third of the total Fiji population. As ethnicity and place of residence (rural/urban) were thought to be the most likely factors to be associated with pneumococcal carriage in each age group, the sampling strategy was proportionate to the ethnic and residential status of Fijians.[1] Included age groups were selected based on those most likely to benefit from the direct and indirect effects of PCV: those with the highest pneumococcal carriage rates, those age-eligible for PCV, and those most likely to transmit pneumococci and be in contact with transmitters. The 12-23 month old age group aligns with peak pneumococcal carriage rates and would be fully vaccinated in later study years. The 2-6 year old age group is an age group responsible for pneumococcal transmission and were expected to have high pneumococcal carriage rates, but the majority would not have received PCV10. 5-8 week old infants represent a vulnerable age group for pneumococcal disease, in which we sought to evaluate potential indirect effects as they are too young to be vaccinated. To assess indirect effects in adults, caregivers were selected as they are the most likely older age group to benefit from indirect effects due to their close interaction with young children.

A convenience sample was used. Communities and health centres were identified based on proximity to ultra-low temperature (ULT) freezers, as carriage samples needed to be stored within 8 h of collection. Recruitment occurred at the two largest health centres located within the Greater Suva area (including surrounding towns): Nausori Health Centre and Valelevu Health Centre and their surrounding communities. A map showing the location of the study area is shown in Supplementary Figure 1.

Two of the largest health centres in Suva were used to recruit infants aged 5-8 weeks attending their immunisation clinics. Caregivers and other age-eligible children were enrolled at the health centres opportunistically. For community recruitment, community health workers identified homes in urban and rural areas with children likely to meet the selection criteria. Eligible household members were recruited. Families who were not home on the day of the study visit were not revisited. Study staff discussed the study with parents/guardians and completed written informed consent.

Recruitment was conducted in the same communities and health centres across study years, with no changes in enrolment procedures. Previous participation in the study was not an exclusion criteria, so some individuals participated in multiple surveys, although this was not recorded. As season may affect carriage, the surveys were conducted during the dry season: September to November 2012; July to November 2013; July to December 2014; and August to November 2015.[2]

It was assumed that no caregivers would have received any doses of PCV10, as the vaccine was not available privately prior to inclusion in the infant immunisation schedule in Fiji, in late October 2012.

The study size was determined based upon an estimated baseline vaccine-type pneumococcal carriage rate of 16% in toddlers.[3] A sample size of 281 would have 90% power at a 5% significance level to show a ~50% reduction in vaccine-type carriage (from 16% to 7%) in the 12-23 month old age group, but as the effect size was anticipated to be lower in the initial years following PCV10 introduction and in unvaccinated age groups, the sample size was increased to 500 for all groups.

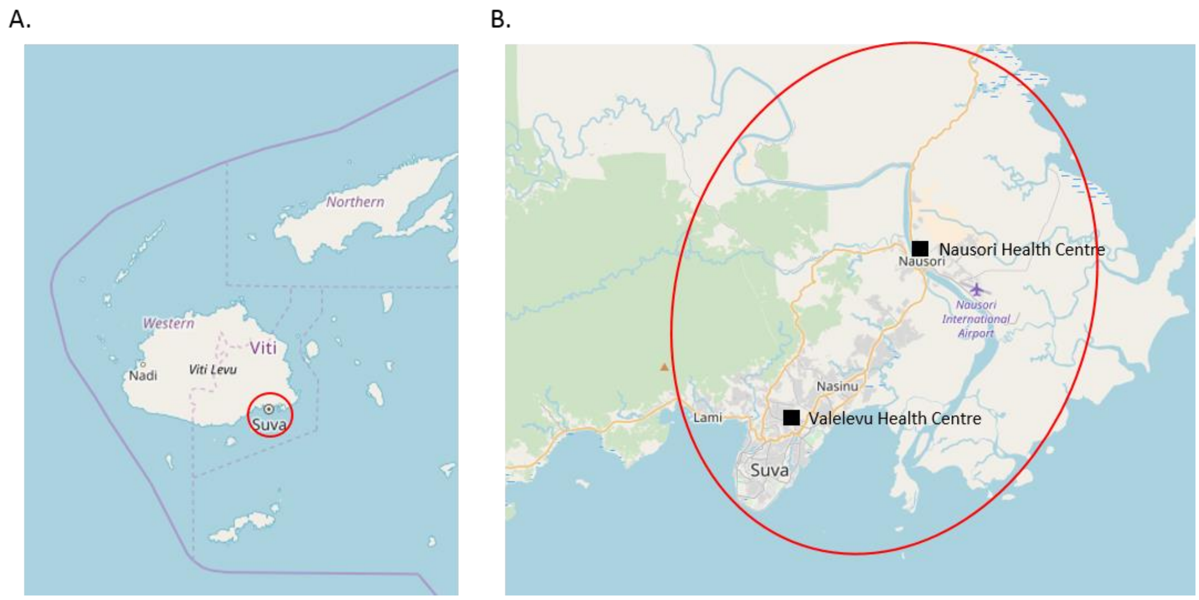

Supplementary Figure 1. Map of the study area. **A.** Location of Suva (circled in red) on the island of Viti Levu. **B.** Greater Suva area (including the surrounding towns of Lami, Nasinu, and Nausori), with the catchment site of potential participants indicated in the red oval. The locations of the two health centres involved in the study are indicated by black boxes. Maps were adapted from https://www.openstreetmap.org/

Laboratory procedures

A flocked, nylon swab was placed in 1 ml skim milk tryptone glucose glycerol media (STGG) immediately following collection. Swabs were stored in a cool box and transported to the Fiji Centre for Communicable Disease Control where they were aliquoted and stored at ULT within 8 h of collection. Samples were shipped to the Murdoch Children's Research Institute (Parkville, Australia) on dry ice and stored at ULT until testing. DNA extraction was conducted on 100 µl of STGG (pelleted by centrifugation for 10 min at 6,000 x g) using a MagNA Pure LC Machine (Roche) using the DNA Isolation Kit III (Bacteria, Fungi) (Roche) following a 30 min incubation at 37°C with 0.16 mg/ml lysostaphin and 3.1 mg/ml lysozyme, and a 2 min incubation at room temperature with 2.5 mg/ml RNase A (Qiagen). Standard curves for quantification were prepared from genomic DNA extracted from reference isolates. qPCR (40 cycles) was conducted in 25 µl reactions containing 2 µl of template DNA on a Stratagene Mx3005 machine using Brilliant III Ultra-Fast qPCR Master Mix (Agilent Technologies) according to the manufacturer's instructions. For pneumococcus, a cycle threshold (Ct) value <35 was considered positive, and Ct values from 35 - 40 were considered equivocal. These equivocal samples were later confirmed by culture. For *H. influenzae*, a Ct value <35 was considered positive, and ≥35 considered negative, as culture was not conducted.

For pneumococcal-positive samples, molecular serotyping using Senti-SP v1.5 microarray (BUGS Bioscience) was conducted following culture amplification as previously described, with minor modifications.[4] Samples were cultured on horse blood agar containing 5 µg/ml of gentamicin and DNA extraction conducted using the QIAcube HT instrument (Qiagen). When only a single α-haemolytic colony grew, it was subcultured prior to DNA extraction for microarray, or in some cases serotyped using latex agglutination.[5] Serotypes 15B and 15C were reported as 15B/C as this serotype is known to interconvert.[6] PCV10 serotypes were defined as the serotypes contained in PCV10 (4, 6B, 9V, 14, 18C, 19F, 23F, 1, 5, and 7F), with all other serotypes including nonencapsulated pneumococci considered non-PCV10 serotypes. Nonencapsulated pneumococci were classified based upon previously described genetic variants (NT1, NT2, NT3a, NT3b, NT4a, NT4b, NT2/NT3b).[7] Serotype-specific density was calculated by multiplying pneumococcal density (determined by *lytA* qPCR) by the relative abundance of the serotype (determined by microarray). All serotyping data were included in the analysis. For example, a swab containing both a PCV10 serotype and a non-PCV10 serotype was considered positive for both PCV10 serotype and non-PCV10 serotype carriage. Any detection of a serotype by microarray was considered positive, regardless of relative abundance or serotype-specific density.

Statistical analysis

Questionnaire results (demographic data) were double data entered into EpiData databases. The two separate EpiData files were validated and corrections made as required using the source document (data collection form). Further cleaning and analyses were conducted in Stata versions 13.1 - 15.1 (StataCorp LLC). Laboratory data was imported to Stata and merged to the demographic Stata file.

Categorical data were summarised as counts and percentages. Continuous data were summarised as medians and interquartile ranges (IQR). Statistical analyses were conducted using GraphPad Prism version 7·03 (GraphPad Software) and Stata versions 14·2 and 15·1 (StataCorp LLC). Month of swab collection was included in the adjusted models for prevalence ratios, as this was found to be associated with pneumococcal carriage in univariable analysis. Month of swab collection was not included in analysis of pneumococcal density as there was no association. Study year was not included in the model for pneumococcal density due to collinearity with vaccination status.

Supplementary Table 1. Prevalence of pneumococcal carriage (overall, PCV10 serotypes, and non-PCV10 serotypes) by age group and year. These data are depicted in Figure 1 in the main paper.

	**Prevalence (95%CI)**			
	**2012**	**2013**	**2014**	**2015**
**All pneumococci**				
5-8w	30·2 (26·1, 34·4)	23·6 (20·0, 27·5)	15·6 (12·5, 19·1)	35·0 (30·8, 39·5)
12-23m	48·6 (44·1, 53·1)	46·7 (42·3, 51·2)	31·4 (27·3, 35·7)	43·6 (39·2, 48·1)
2-6y	52·7 (48·3, 57·1)	45·7 (41·4, 50·1)	33·3 (29·2, 37·6)	43·3 (39·0, 47·8)
caregivers	10·4 (7·9, 13·4)	13·1 (10·3, 16·3)	6·6 (4·6, 9·1)	7·8 (5·7, 10·5)
**PCV10 serotypes**				
5-8w	9·6 (7·1, 12·6)	5·9 (4·0, 8·4)	3·5 (2·0, 5·5)	5·8 (3·9, 8·3)
12-23m	22·3 (18·7, 26·2)	18·8 (15·5, 22·5)	8·0 (5·7, 10·7)	7·3 (5·2, 9·9)
2-6y	21·7 (18·2, 25·6)	14·8 (11·8, 18·1)	7·6 (5·4, 10·3)	9·1 (6·7, 11·9)
caregivers	2·4 (1·2, 4·1)	3·3 (2·0, 5·3)	1·6 (0·7, 3·0)	0·8 (0·2, 2·0)
**Non-PCV10 serotypes**				
5-8w	20·8 (17·3, 24·7)	17·8 (14·6, 21·4)	12·0 (9·3, 15·2)	30·1 (26·1, 34·5)
12-23m	30·4 (26·4, 34·7)	35·1 (30·9, 39·4)	26·1 (22·3, 30·2)	38·5 (34·1, 42·9)
2-6y	39·7 (35·4, 44·1)	37·7 (33·5, 42·0)	27·5 (23·7, 31·7)	37·3 (33·1, 41·7)
caregivers	8·1 (5·9, 10·8)	10·0 (7·6, 13·0)	5·4 (3·6, 7·7)	6·7 (4·7, 9·2)

Supplementary Table 2. Carriage prevalence for pneumococcal carriage (overall, PCV10 serotypes, and non-PCV10 serotypes) by age group and year, analysed separately for participants of Fijians of Indian Descent and iTaukei ethnicity. These data are depicted in Figure 2 in the main paper.

	Carriers/total<br>Carriage prevalence (95%CI)			
	**2012**	**2013**	**2014**	**2015**
**All pneumococci, Fijians of Indian Descent**				
5-8w	46/195	22/197	11/205	23/192
	23·6 (17·8, 30·2)	11·2 (7·1, 16·4)	5·4 (2·7, 9·4)	12·0 (7·8, 17·4)
12-23m	69/196	61/202	33/206	45/196
	35·2 (28·5, 42·3)	30·2 (24·0, 37·0)	16·0 (11·3, 21·7)	23·0 (17·3, 29·5)
2-6y	55/196	32/208	39/203	35/206
	28·1 (21·9, 34·9)	15·4 (10·8, 21·0)	19·2 (14·0, 25·3)	17·0 (12·1, 22·8)
caregivers	5/196	9/202	6/215	5/203
	2·5 (0·8, 5·8)	4·4 (2·1, 8·3)	2·8 (1·0, 6·0)	2·5 (0·8, 5·6)
**All pneumococci, iTaukei**				
5-8w	102/297	98/309	66/287	148/292
	34·3 (29·0, 40·0)	31·7 (26·6, 37·2)	23·0 (18·2, 28·3)	50·7 (44·8, 56·6)
12-23m	170/299	174/301	122/288	171/298
	56·9 (51·0, 62·5)	57·8 (52·0, 63·4)	42·4 (36·6, 48·3)	57·4 (51·6, 63·1)
2-6y	213/312	201/305	128/297	185/301
	68·3 (62·8, 73·4)	65·9 (60·3, 71·2)	43·1 (37·4, 48·9)	61·5 (55·7, 67·0)
caregivers	48/310	58/309	28/296	34/304
	15·5 (11·6, 20·0)	18·8 (14·6, 23·4)	9·5 (6·4, 13·4)	11·2 (7·9, 15·3)
**PCV10 serotypes, Fijians of Indian Descent**				
5-8w	16/193	7/195	1/204	7/192
	8·3 (4·8, 13·1)	3·6 (1·5, 7·3)	0·5 (0·0, 2·7)	3·6 (1·5, 7·4)
12-23m	291/196	25/202	4/205	11/196
	14·8 (10·1, 20·5)	12·4 (8·2, 17·7)	2·0 (0·5, 4·9)	5·6 (2·8, 9·8)
2-6y	18/193	5/208	11/202	9/205
	9·3 (5·6, 14·3)	2·4 (0·8, 5·5)	5·4 (2·7, 9·5)	4·4 (2·0, 8·2)
caregivers	0/195	0/202	2/215	2/203
	0·0 (0·0, 1·9)[1]	0·0 (0·0, 1·9)[1]	0·9 (0·1, 3·3)	1·0 (0·1, 3·5)
**PCV10 serotypes, iTaukei**				
5-8w	30/285	23/307	16/285	21/285
	10·5 (7·2, 14·7)	7·5 (4·8, 11·1)	5·6 (3·2, 9·0)	7·4 (4·6, 11·0)
12-23m	79/294	69/297	35/285	25/297
	26·9 (21·9, 32·3)	23·2 (18·6, 28·5)	12·3 (8·7, 16·7)	8·4 (5·5, 12·2)
2-6y	91/310	70/304	27/295	37/298
	29·4 (24·3, 34·8)	23·0 (18·4, 28·2)	9·1 (6·1, 13·0)	12·4 (8·9, 16·8)
caregivers	12/309	17/307	6/296	2/302
	3·9 (2·0, 6·7)	5·5 (3·3, 8·7)	2·0 (0·8, 4·4)	0·7 (0·1, 2·4)
**Non-PCV10 serotypes, Fijians of Indian Descent**				
5-8w	28/193	13/195	9/204	17/192
	14·5 (9·9, 20·3)	6·7 (3·6, 11·1)	4·4 (2·0, 8·2)	8·8 (5·2, 13·8)
12-23m	41/196	42/202	28/205	34/196
	20·9 (15·4, 27·3)	20·8 (15·4, 27·0)	13·7 (9·3, 19·1)	17·3 (12·3, 23·4)
2-6y	35/193	27/208	28/202	26/205
	18·1 (13·0, 24·3)	13·0 (8·7, 18·3)	13·9 (9·4, 19·4)	12·7 (8·4, 18·0)
caregivers	4/195	9/202	4/215	3/203

	2·0 (0·6, 5·2)	4·4 (2·1, 8·3)	1·9 (0·5, 4·7)	1·5 (0·3, 4·2)
**Non-PCV10 serotypes, iTaukei**				
5-8w	71/285	76/307	50/285	128/285
	24·9 (20·0, 30·4)	24·8 (20·0, 30·0)	17·5 (13·3, 22·5)	44·9 (39·0, 50·9)
12-23m	108/294	133/297	100/285	156/297
	36·7 (31·2, 42·5)	44·8 (39·0, 50·6)	35·1 (29·6, 40·9)	52·5 (46·7, 58·3)
2-6y	165/310	165/304	109/295	162/298
	53·2 (47·5, 58·9)	54·3 (48·5, 60·0)	37·0 (31·4, 42·7)	54·4 (48·5, 60·1)
caregivers	37/309	42/307	24/296	30/302
	12·0 (8·6, 16·1)	13·7 (10·0, 18·0)	8·1 (5·3, 11·8)	9·9 (6·8, 13·9)

[1]One-sided 95%CI

Supplementary Table 3. Unadjusted (PR) and adjusted prevalence ratios (aPR) for pneumococcal carriage (overall, PCV10 serotypes, and non-PCV10 serotypes) and compared with 2012 (pre-PCV10) by age group and year, analysed separately for participants of Fijians of Indian Descent and iTaukei ethnicity.

	Prevalence ratios compared with 2012 (95%CI)					
	2013		2014		2015	
	PR	aPR[1]	PR	aPR[1]	PR	aPR[1]
**All pneumococci, Fijians of Indian Descent**						
5-8w	0·47 (0·30, 0·76)	0·29 (0·16, 0·52)	0·23 (0·12, 0·43)	0·29 (0·13, 0·62)	0·51 (0·32, 0·80)	0·44 (0·27, 0·71)
12-23m	0·86 (0·65, 1·14)	0·55 (0·42, 0·72)	0·46 (0·32, 0·66)	0·40 (0·27, 0·59)	0·65 (0·47, 0·90)	0·56 (0·41, 0·77)
2-6y	0·55 (0·37, 0·81)	0·53 (0·31, 0·88)	0·68 (0·48, 0·98)	0·76 (0·49, 1·18)	0·60 (0·42, 0·88)	0·64 (0·42, 0·99)
caregivers	1·75 (0·60, 5·12)	1·82 (0·46, 7·17)	1·09 (0·34, 3·53)	1·27 (0·33, 4·86)	0·96 (0·28, 3·28)	1·00 (0·25, 3·98)
**All pneumococci, iTaukei**						
5-8w[2]	0·92 (0·74, 1·16)	1·17 (0·89, 1·55)	0·67 (0·51, 0·87)	0·89 (0·64, 1·23)	1·48 (1·22, 1·79)	1·68 (1·34, 2·11)
12-23m	1·02 (0·89, 1·17)	0·95 (0·82, 1·09)	0·74 (0·63, 0·88)	0·76 (0·64, 0·90)	1·01 (0·88, 1·16)	1·00 (0·86, 1·16)
2-6y[2]	0·96 (0·86, 1·08)	0·94 (0·80, 1·10)	0·63 (0·54, 0·73)	0·67 (0·57, 0·79)	0·90 (0·80, 1·01)	0·98 (0·86, 1·12)
caregivers	1·21 (0·86, 1·72)	1·13 (0·72, 1·76)	0·61 (0·39, 0·95)	0·63 (0·39, 1·03)	0·72 (0·48, 1·09)	0·77 (0·50, 1·19)
**PCV10 serotypes, Fijians of Indian Descent**						
5-8w	0·43 (0·18, 1·03)	0·29 (0·10, 0·87)	0·06 (0·01, 0·44)	0·09 (0·01, 0·75)	0·44 (0·18, 1·04)	0·36 (0·14, 0·92)
12-23m	0·84 (0·51, 1·38)	0·31 (0·18, 0·54)	0·13 (0·05, 0·37)	0·11 (0·04, 0·30)	0·38 (0·20, 0·74)	0·29 (0·15, 0·56)
2-6y	0·26 (0·10, 0·68)	0·25 (0·07, 0·87)	0·58 (0·28, 1·2)	0·72 (0·29, 1·77)	0·47 (0·22, 1·02)	0·41 (0·16, 1·03)
caregivers	Not determined[3]	Not determined	Not determined	Not determined	Not determined	Not determined
**PCV10 serotypes, iTaukei**						
5-8w	0·71 (0·42, 1·20)	0·51 (0·26, 0·99)	0·53 (0·30, 0·96)	0·39 (0·18, 0·84)	0·70 (0·41, 1·19)	0·58 (0·31, 1·09)
12-23m	0·86 (0·65, 1·14)	0·65 (0·47, 0·89)	0·46 (0·32, 0·66)	0·45 (0·30, 0·66)	0·31 (0·21, 0·32)	0·33 (0·21, 0·52)
2-6y	0·78 (0·60, 1·03)	0·62 (0·42, 0·90)	0·31 (0·21, 0·46)	0·30 (0·19, 0·46)	0·42 (0·30, 0·60)	0·47 (0·32, 0·68)
caregivers	1·42 (0·69, 2·93)	1·16 (0·42, 3·17)	0·52 (0·20, 1·37)	0·51 (0·16, 1·59)	0·17 (0·04, 0·76)	0·21 (0·04, 0·98)
**Non-PCV10 serotypes, Fijians of Indian Descent**						
5-8w	0·46 (0·24, 0·86)	0·31 (0·14, 0·68)	0·30 (0·15, 0·63)	0·49 (0·21, 1·16)	0·61 (0·35, 1·08)	0·56 (0·31, 1·02)
12-23m	1·00 (0·68, 1·46)	0·68 (0·41, 1·13)	0·65 (0·42, 1·01)	0·59 (0·36, 0·97)	0·83 (0·55, 1·25)	0·74 (0·48, 1·14)
2-6y	0·71 (0·45, 1·14)	0·64 (0·35, 1·18)	0·76 (0·48, 1·20)	0·79 (0·45, 1·37)	0·70 (0·44, 1·12)	0·78 (0·46, 1·33)
caregivers	2·17 (0·68, 6·94)	2·03 (0·42, 9·75)	0·91 (0·23, 3·56)	0·89 (0·17, 4·58)	0·72 (0·16, 3·18)	0·64 (0·12, 3·40)
**Non-PCV10 serotypes, iTaukei**						
5-8w	0·99 (0·75, 1·32)	1·55 (1·11, 2·16)	0·70 (0·51, 0·97)	1·13 (0·76, 1·67)	1·80 (1·42, 2·29)	2·23 (1·70, 2·92)
12-23m	1·22 (1·00, 1·48)	1·14 (0·92, 1·42)	0·96 (0·77, 1·19)	0·98 (0·78, 1·25)	1·42 (1·19, 1·72)	1·42 (1·16, 1·74)
2-6y	1·02 (0·88, 1·18)	1·12 (0·92, 1·36)	0·69 (0·58, 0·83)	0·81 (0·66, 0·99)	1·02 (0·88, 1·18)	1·19 (1·01, 1·40)
caregivers	1·14 (0·76, 1·73)	1·13 (0·68, 1·89)	0·68 (0·41, 1·10)	0·73 (0·42, 1·25)	0·83 (0·53, 1·31)	0·89 (0·55, 1·44)

[1]The following variables were adjusted for each age group: 5–8 weeks: two or more children under five years in the household [multiple <5y], symptoms of upper respiratory tract infection [URTI], poverty, mode of delivery, breastfeeding status, month of swab collection; 12–23 months: residential location, multiple <5y, URTI, poverty, and month of swab collection; 2–6 years: residential location, multiple <5y, exposure to household cigarette smoke, URTI, poverty, and month of swab collection; caregivers: sex, multiple <5y, URTI, poverty, and month of swab collection.

[2]Binomial regression model data did not converge, so a Poisson model was used to estimate adjusted prevalence ratios.

[3]Prevalence ratios not determined as 2012 prevalence was 0%.

Supplementary Table 4. Proportion of total pneumococci identified in nasopharyngeal carriage samples that belonged to PCV10 serotypes, shown by age group and year.

	**2012**	**2013**	**P value[1]**	**2014**	**P value**	**2015**	**P value**
5-8w	48/160 (30·0%)	31/135 (23·0%)	0·174	17/86 (20%)	0·083	30/201 (14·9%)	0·0005
12-23m	116/296 (39·2%)	101/330 (30·6%)	0·0243	42/192 (21·9%)	<0·0001	36/281 (12·8%)	<0·0001
2-6y	126/384 (32·8%)	82/346 (23·7%)	0·0065	40/208 (19·2%)	0·0004	50/298 (16·7%)	<0·0001
caregivers	12/59 (20%)	17/71 (24%)	0·623	8/37 (22%)	0·880	4/40 (10%)	0·170

[1]Compared with 2012 (chi-squared test)

A.

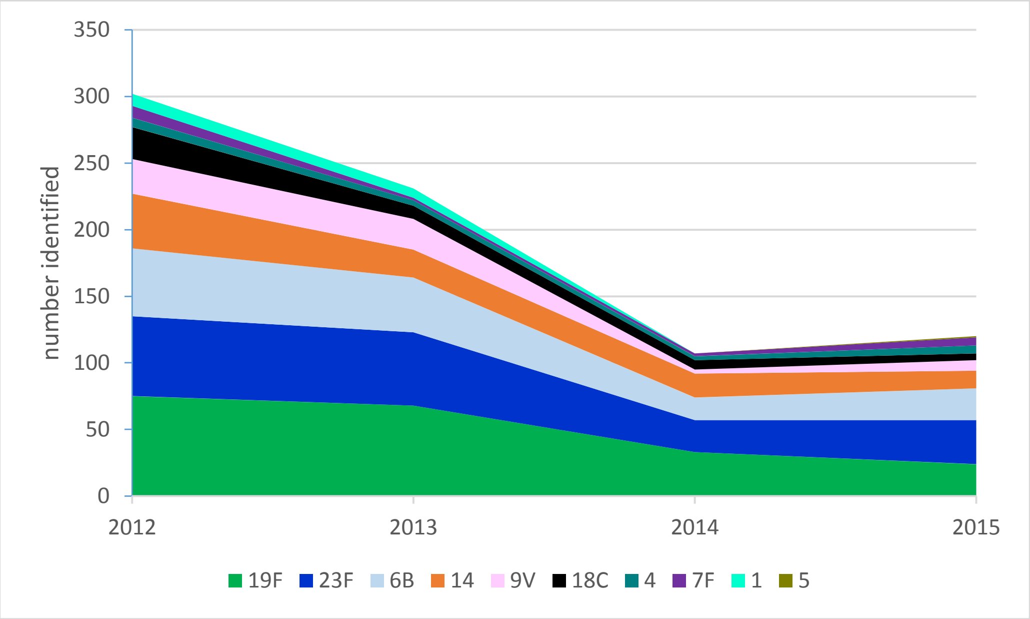
number identified
350
300
250
200
150
100
50
0
2012
2013
2014
2015
19F
23F
6B
14
9V
18C
4
7F
1
5

B.

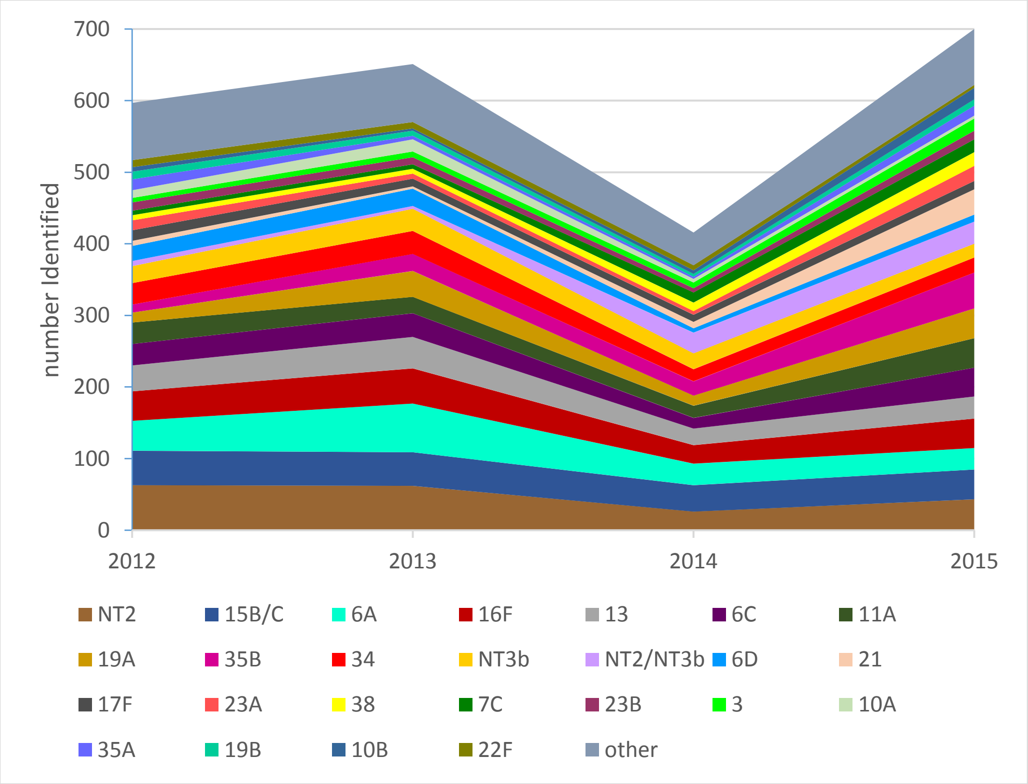
number Identified
700
600
500
400
300
200
100
0
2012
2013
2014
2015
NT2
15B/C
6A
16F
13
6C
11A
19A
35B
34
NT3b
NT2/NT3b
6D
21
17F
23A
38
7C
23B
3
10A
35A
19B
10B
22F
other

C.

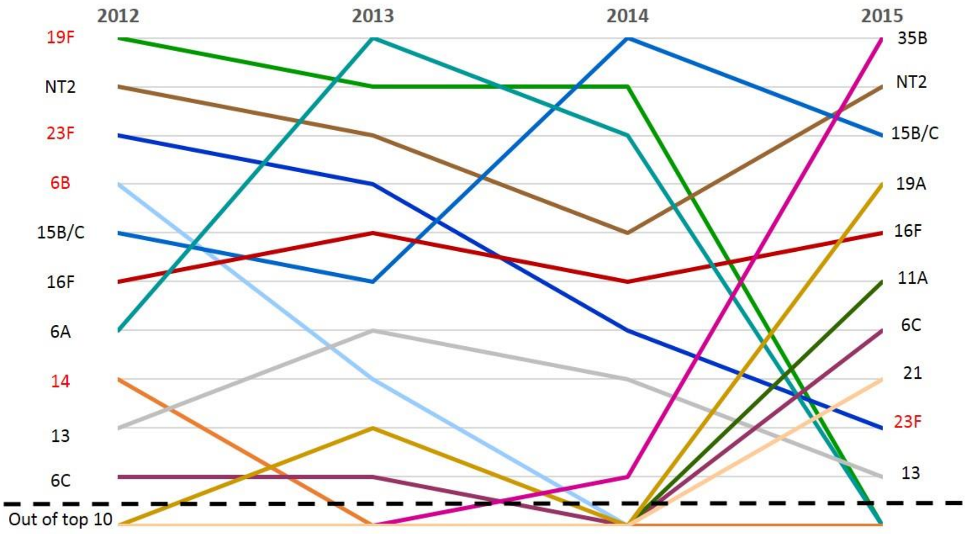

Supplementary Figure 2. A. Stacked graph showing the number of individual PCV10 serotypes identified each year from all participants. **B.** Stacked graph showing the number of non-PCV10 serotypes identified each year from all participants. The 25 most common non-PCV10 serotypes are shown individually with the remainder grouped as other. **C**. Ranking of the top 10 most common serotypes by year, with the most common listed at the top. PCV10 serotypes are indicated in red font.

Supplementary Table 5. Serotype-specific carriage data for paediatric age groups. Data correspond to Figure 3 in the main paper. All PCV10 serotypes are included in the table, as well as vaccine-related serotypes 6A and 19A and additional non-vaccine types that significantly increased (p<0·05 2012 vs 2015).

	**2012** n (%)	**2013** n (%)	**2014** n (%)	**2015** n (%)	**P value[1]**
**5-8w**					
Number of samples[2]	480	505	491	481	
PCV10 serotypes					
19F	12 (2·50)	14 (2·77)	3 (0·61)	8 (1·66)	0·364
6B	14 (2·92)	7 (1·39)	2 (0·41)	5 (1·04)	0·0366
23F	5 (1·04)	7 (1·39)	7 (1·43)	7 (1·46)	0·564
14	3 (0·63)	1 (0·20)	3 (0·61)	2 (0·42)	0·652
7F	3 (0·63)	0 (0·00)	1 (0·20)	3 (0·62)	0·998
9V	3 (0·63)	0 (0·00)	1 (0·20)	2 (0·42)	0·652
18C	4 (0·83)	0 (0·00)	0 (0·00)	1 (0·21)	0·178
4	3 (0·63)	1 (0·20)	0 (0·00)	2 (0·42)	0·652
1	1 (0·21)	1 (0·20)	0 (0·00)	0 (0·00)	0·317
5	0 (0·00)	0 (0·00)	0 (0·00)	0 (0·00)	-
Non-PCV10 serotypes					
6A	5 (1·04)	9 (1·78)	4 (0·81)	8 (1·66)	0·404
19A	2 (0·42)	5 (0·99)	3 (0·61)	10 (2·08)	0·0203
35B	2 (0·42)	5 (0·99)	5 (1·02)	12 (2·49)	0·0072
21	3 (0·63)	1 (0·20)	3 (0·61)	14 (2·91)	0·0072
**12-23m**					
Number of samples	493	499	490	494	
PCV10 serotypes					
19F	36 (7·30)	27 (5·41)	13 (2·65)	10 (2·02)	<0·0001
6B	18 (3·65)	20 (4·01)	5 (1·02)	7 (1·42)	0·0255
23F	27 (5·48)	25 (5·01)	11 (2·24)	11 (2·23)	0·0080
14	15 (3·04)	9 (1·80)	5 (1·02)	2 (0·40)	0·0004
7F	2 (0·41)	1 (0·20)	1 (0·20)	1 (0·20)	0·562
9V	12 (2·43)	12 (2·40)	0 (0·00)	3 (0·61)	0·0190
18C	6 (1·22)	5 (1·00)	6 (1·22)	1 (0·20)	0·058
4	0 (0·00)	0 (0·00)	1 (0·20)	0 (0·00)	-
1	0 (0·00)	2 (0·40)	0 (0·00)	0 (0·00)	-
5	0 (0·00)	0 (0·00)	0 (0·00)	1 (0·20)	0·381
Non-PCV10 serotypes					
6A	19 (3·85)	23 (4·61)	10 (2·04)	15 (3·04)	0·481
19A	5 (1·01)	18 (3·61)	3 (0·61)	14 (2·83)	0·0375
35B	4 (0·81)	13 (2·61)	7 (1·43)	18 (3·64)	0·0026
21	0 (0·00)	1 (0·20)	3 (0·61)	11 (2·23)	0·0009
16F	10 (2·03)	18 (3·61)	9 (1·84)	21 (4·25)	0·0453
NT2/NT3b	0 (0·00)	1 (0·20)	8 (1·63)	10 (2·02)	0·0015
**2-6 y**					
Number of samples	511	515	501	506	
PCV10 serotypes					
19F	26 (5·09)	25 (4·85)	13 (2·59)	6 (1·19)	0·0004
6B	18 (3·52)	11 (2·14)	10 (2·00)	12 (2·37)	0·278
23F	26 (5·09)	15 (2·91)	6 (1·20)	13 (2·57)	0·0365
14	21 (4·11)	11 (2·14)	7 (1·40)	7 (1·38)	0·0079
7F	4 (0·78)	0 (0·00)	0 (0·00)	2 (0·40)	0·420
9V	9 (1·76)	10 (1·94)	1 (0·20)	3 (0·59)	0·084
18C	12 (2·35)	5 (0·97)	1 (0·20)	3 (0·59)	0·0202
4	3 (0·59)	3 (0·58)	2 (0·40)	4 (0·79)	0·695
1	7 (1·37)	2 (0·39)	0 (0·00)	0 (0·00)	0·0082
5	0 (0·00)	0 (0·00)	0 (0·00)	0 (0·00)	-
Non-PCV10 serotypes					
6A	17 (3·33)	33 (6·41)	13 (2·59)	7 (1·38)	0·0412
19A	7 (1·37)	12 (2·33)	8 (1·60)	15 (2·96)	0·081
35B	5 (0·98)	6 (0·98)	8 (1·60)	18 (3·56)	0·0057

[1]p<0·05 2012 vs 2015, chi-squared test

[2]Samples that were qPCR positive (Ct value <35) and culture negative were not serotyped, and excluded from this analysis. Therefore, the number of samples is lower than the number of participants shown in Table 1 of the main paper.

Acknowledgements

We thank the study participants and their families, as well as the health workers and community leaders involved. We thank the Fiji Ministry of Health and Medical Services for providing the clinic, office and laboratory space. We thank the New Vaccine Evaluation Project staff, particularly Mereadani Malimali and Kathryn Bright, for help with enrolment and sample collection, and Maha Habib, Jenna Smyth, Regina Oakley, Barbara Porter, and Ahmed Alamrousi for assistance with laboratory analyses. We acknowledge Rachel Devi and Adam Jenney for assistance with study design. We thank the Fiji Health Sector Support Project for their support and enabling environment. This project was supported by the Bill & Melinda Gates Foundation (OPP126272 and OPP1084341) and the Victorian Government's Operational Infrastructure Support Program, and the Department of Foreign Affairs and Trade of the Australian Government and Fiji Health Sector Support Program (FHSSP). FHSSP was implemented by Abt JTA on behalf of the Australian Government. FMR and CS are supported by fellowships from the Australian National Health and Medical Research Council, and CS was also supported by a veski Inspiring Women Fellowship. EFGN is supported by an Australian Government Research Training Program Scholarship.

References

1. Russell FM, Carapetis JR, Ketaiwai S, Kunabuli V, Taoi M, Biribo S, et al. Pneumococcal nasopharyngeal carriage and patterns of penicillin resistance in young children in Fiji. Ann Trop Paediatr. 2006;**26**:187-97.
2. Abdullahi O, Nyiro J, Lewa P, Slack M, Scott JAG. The descriptive epidemiology of *Streptococcus pneumoniae* and *Haemophilus influenzae* nasopharyngeal carriage in children and adults in Kilifi District, Kenya. Pediatr Infect Dis J. 2008;**27**(1):59-64.
3. Russell FM, Carapetis JR, Satzke C, Tikoduadua L, Waqatakirewa L, Chandra R, et al. Pneumococcal nasopharyngeal carriage following reduced doses of a 7-valent pneumococcal conjugate vaccine and a 23-valent pneumococcal polysaccharide vaccine booster. Clin Vaccine Immunol. 2010;17:1970-6.
4. Satzke C, Dunne EM, Porter BD, Klugman KP, Mulholland EK, PneuCarriage project group. The PneuCarriage Project: a multi-centre comparative study to identify the best serotyping methods for examining pneumococcal carriage in vaccine evaluation studies. *PLoS Med* 2015; **12**(11):e1001903
5. Porter BD, Ortika BD, Satzke C. Capsular serotyping of *Streptococcus pneumoniae* by latex agglutination. *J Vis Exp* 2014; (91): 51747.
6. van Selm S, van Cann LM, Kolkman MAB, van der Zeijst BAM, van Putten JPM. Genetic basis for the structural difference between *Streptococcus pneumoniae* serotype 15B and 15C capsular polysaccharides. *Infect Immun* 2003; **71**(11): 6192, 8.
7. Salter SJ, Hinds J, Gould KA, et al. Variation at the capsule locus, *cps*, of mistyped and non-typable *Streptococcus pneumoniae* isolates. *Microbiol* 2012; **158**(6): 1560, 9.
